# Utilizing a sutureless valve for prosthetic valve endocarditis after aortic root replacement

**DOI:** 10.1186/s40792-024-01977-9

**Published:** 2024-07-30

**Authors:** Yukihiro Hayatsu, Masaaki Naganuma, Hayate Nomura, Kazuhiro Yamaya, Masaki Hata

**Affiliations:** Department of Cardiovascular Surgery, Sendai Kosei Hospital, 1-20 Tsutsumidori Amamiyamachi, Aoba Ward, Sendai City, Miyagi 981-0914 Japan

**Keywords:** Redo operation, Sutureless valve, Aortic root replacement

## Abstract

**Background:**

Reoperation following aortic root replacement is associated with significantly high operative mortality. Etiologies related to infection are known to increase the operative mortality rate more than other etiologies. In such a clinical setting, a sutureless valve could lower the operative mortality by shortening the cardiac arrest and the operative time.

**Case presentation:**

A 61-year-old male underwent emergent aortic root and total arch replacement with an open stent graft for acute type-A aortic dissection. A bioprosthetic valve was employed for aortic root replacement using the double-sewing ring technique. A fungal infection by *Candida parapsilosis* was postoperatively detected and improved with intravenous antifungal drug administration. However, he developed congestive heart failure one year later, and the blood cultures turned positive repeatedly for *Candida parapsilosis*. The prosthetic valve infection was suspected upon identifying vegetation on the bioprosthetic valve through transthoracic echocardiography. The computed tomography scan and operative findings confirmed that the infection was localized on the prosthetic valve. Consequently, the infected valve was removed without a vascular conduit, and a sutureless valve was implanted. The postoperative course was uneventful, without any evidence of recurrent fungal infection, and the patient was discharged on postoperative day 28.

**Conclusions:**

Deploying a sutureless valve can facilitate a more straightforward and minimally invasive redo procedure. Preoperative computed tomography can predict the valve size, which is the key to implanting a sutureless valve successfully after the modified Bentall procedure.

## Background

Aortic root surgery has been safely performed since the introduction of the Bentall procedure [1] in 1968. However, a redo aortic root operation still poses a particular operative risk, ranging from 5.0% to 12.1% [2–4], and varies depending on the etiology of the redo intervention. Most published data have shown that endocarditis is a risk factor for the deterioration of early and late outcomes after redo aortic root replacement.

Various types of aortic root replacement have been documented, with the prosthetic valve fixed to the aortic annulus in both the original and modified Bentall procedures [1, 5]. Therefore, a reoperation following such types of aortic root replacement would oblige us to truly intervene within the aortic root, which requires the dissection of tight adhesions without damaging the coronary ostium in a deep operative field. In contrast, a self-assembled composite graft with the prosthetic valve remaining unfixed to the annulus [6–8] confers a notable advantage in facilitating reoperations. In the event of prosthetic valve failure, only the failed prosthetic valve has to be removed, and a new prosthetic valve can be easily fixed on the annulus or vascular graft without requiring aortic root intervention. A sutureless valve can also be applied to lower the operative risk by shortening the operative time, even in a difficult operative field. Here, we describe a case of sutureless valve use for reoperation following an aortic root replacement.

## Case presentation

**Fig. 1:**
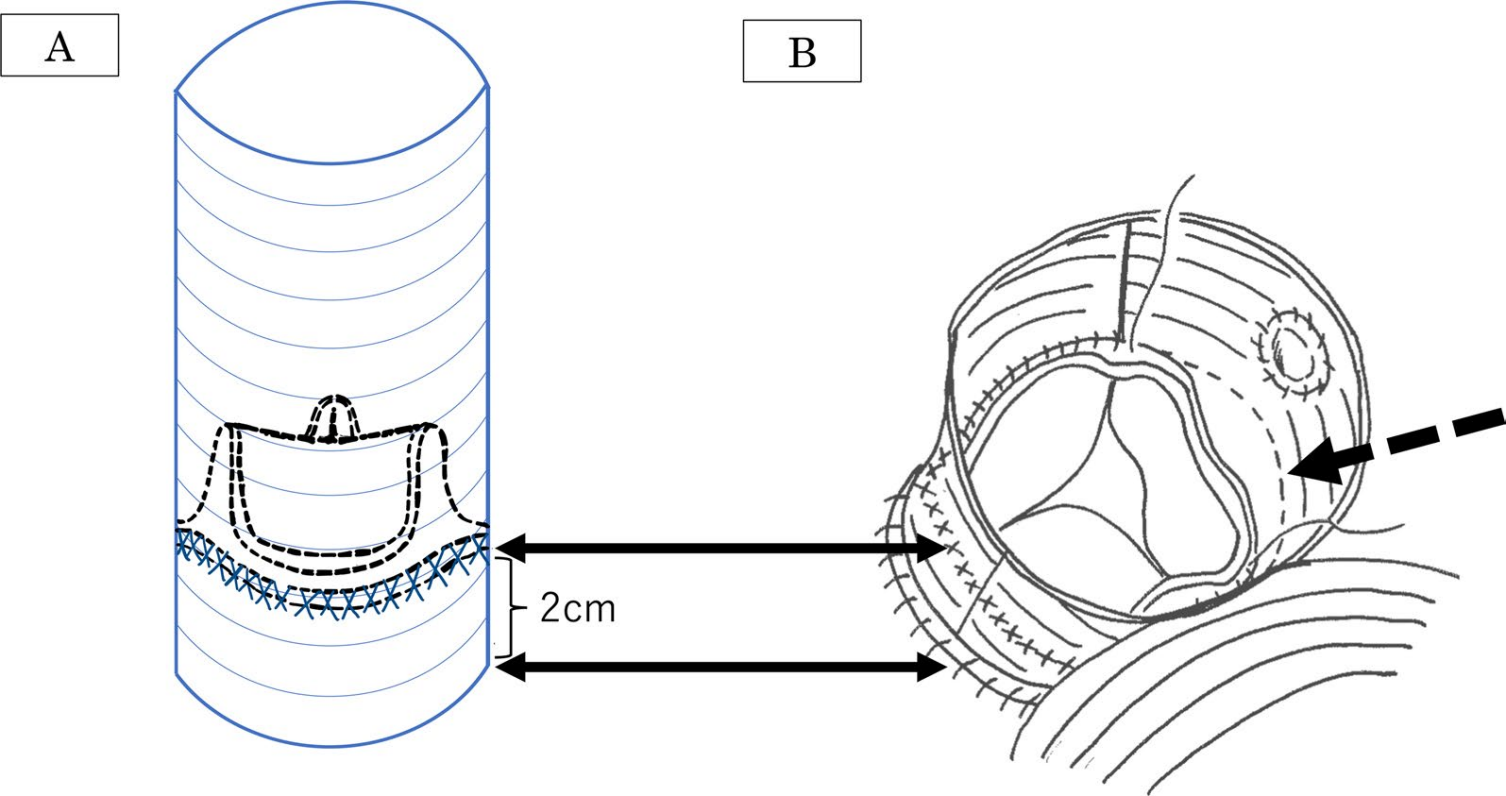
Scheme of the composite graft and the extraction of a bioprosthetic valve from the composite graft. **A** Scheme of the composite graft with a bioprosthetic valve. A bioprosthetic valve is sewn 2 cm above the bottom of the vascular graft with external continuous sutures (each arrows correspond and indicate the suture lines in **B**). **B** Extraction of a bioprosthetic valve. A bioprosthetic valve could be easily extracted by cutting sutures that fixed it to the vascular graft without dissecting adhesion (black arrow)

A 61-year-old male with a history of hypertension was referred to our hospital for syncope after severe back pain. Enhanced computed tomography (CT) revealed type-A acute aortic dissection, necessitating emergency surgery. Intraoperatively, an intimal tear was located immediately above the left coronary ostium, extending into the right coronary artery. Consequently, aortic root replacement was performed with a composite graft assembled with an Inspiris Resilia aortic valve size 23 mm (Edwards Lifesciences LLC, Irvine, CA, USA) and a Gelweave graft size 26 mm (Vascutek, Renfrewshire, Scotland, UK) (Fig. 1A). In addition, total arch replacement, and coronary artery bypass grafting (CABG) of the right coronary artery (RCA) were accomplished. Due to prolonged aortic cross-clamp time, the patient could not be weaned off cardiopulmonary bypass (CPB), and venoarterial extracorporeal membrane oxygenation (VA-ECMO) was initiated to terminate CPB. Fortunately, cardiac function improved, and VA-ECMO was terminated on postoperative day (POD) 3. However, blood cultures revealed a fungal infection (*Candida parapsilosis*) on POD 30. The patient had a low-grade fever and increased inflammatory reaction, but there was no evidence of vascular graft or prosthetic valve infection on any diagnostic modalities. A loading dose of 800 mg of fosfluconazole (F-FLCZ) was administered for two days, followed by 400 mg of F-FLCZ as a maintenance dose for preventing infection of artificial materials. Despite the administration of F-FLCZ, *Candida parapsilosis* had been positive. Therefore, 150 mg of Micafungin sodium hydrate (MCFG) was additionally administered. After that, the blood culture turned negative, and the patient was discharged with 100 mg of fluconazole.

**Fig. 2:**
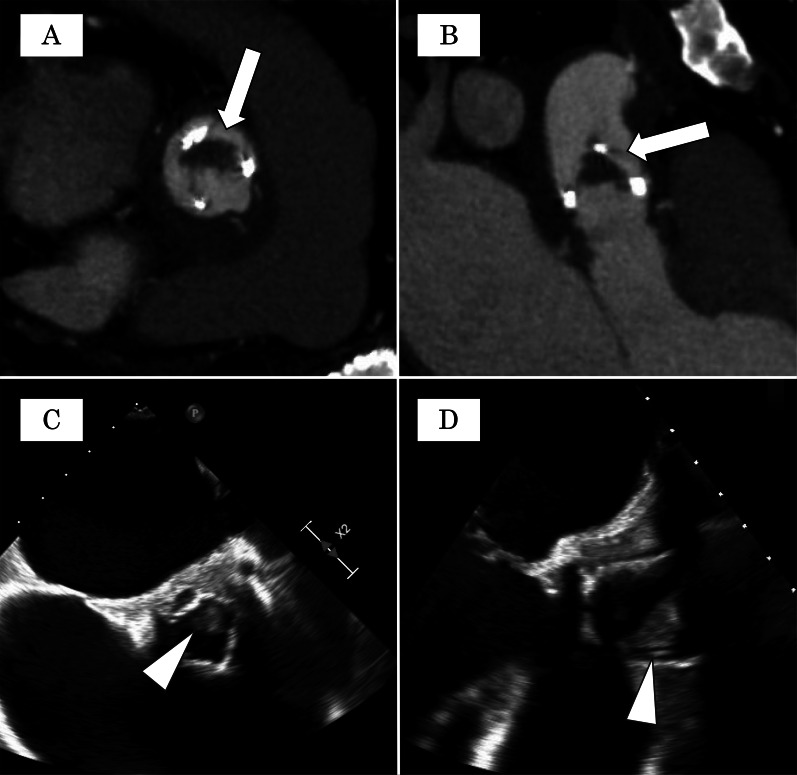
Preoperative echocardiography. The white arrow points to a low-density mass on the leaflet by CT scan, suggesting vegetation (**A** short axis view, **B** long axis view). The arrowhead indicates a low echoic and mobile mass by transthoracic echocardiography (**C** short axis view, **D** long axis view)

One year after discharge, the patient was again hospitalized for fever and dyspnea. The fungal species *Candida parapsilosis* was again detected in blood cultures. Enhanced CT and transesophageal echocardiography (TEE) revealed a bulky, low-density mass on the prosthetic valve, suggesting prosthetic valve endocarditis (Fig. 2A–D). Despite one month of guideline-directed antifungal therapy, *Candida parapsilosis* remained positive in blood cultures, and symptoms of heart failure gradually became apparent. Hence, a reoperation was planned.

**Fig. 3:**
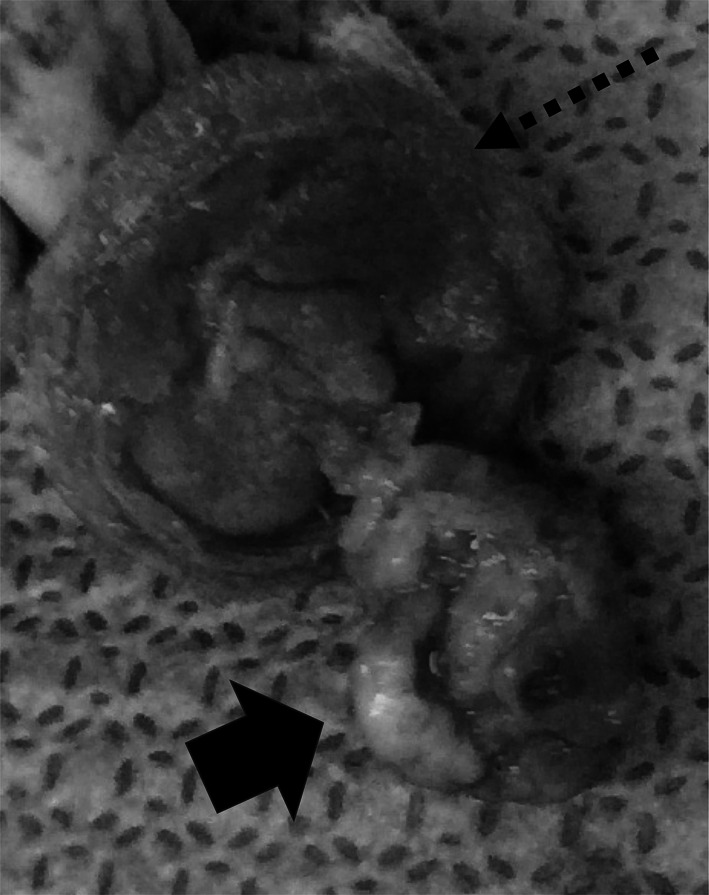
Infected bioprosthetic valve. The infected prosthetic valve was extracted without damaging the sewn cuff (dotted arrow). Vegetation attached to the leaflet on the left ventricular side can be observed (black arrow)

A redo median sternotomy was performed, and CPB was initiated with the right axillary and the right common femoral arterial perfusion and bicaval drainage. The vascular graft was observed to adhere tightly to the surrounding tissue and was not macroscopically infected. Before cross-clamping the vascular graft, CABG of the RCA was performed with a saphenous vein graft (SVG) because the prior SVG was occluded on the preoperative CT scan. Antegrade cardioplegia was selectively administered via the SVG to the RCA. The prosthetic valve was exposed upon transection of the vascular graft, and a bulky vegetation was observed on its leaflet (Fig. 3). The infected prosthetic valve was then carefully removed without damaging the vascular graft and the left coronary ostium (Fig. 1B). The vascular graft appeared intact from the inner side and was negative for bacteria on intraoperative rapid Gram staining. Therefore, we performed aortic valve replacement (AVR) using the Perceval™ valve (Corcym, Austin, TX, USA). Size S was selected after meticulous irrigation around the annulus and vascular graft. The first guiding suture was placed on the annulus under the left coronary ostium, and the other two guiding sutures were placed separately at 120° from the left coronary ostium. The Perceval^TM^ valve was then carefully deployed, followed by ballooning at 4 atm for 30 s and an additional 15 s at 5 atm. After completion of the proximal anastomosis of the SVG and distal anastomosis of the vascular graft, the cross-clamp was released.

**Fig. 4:**
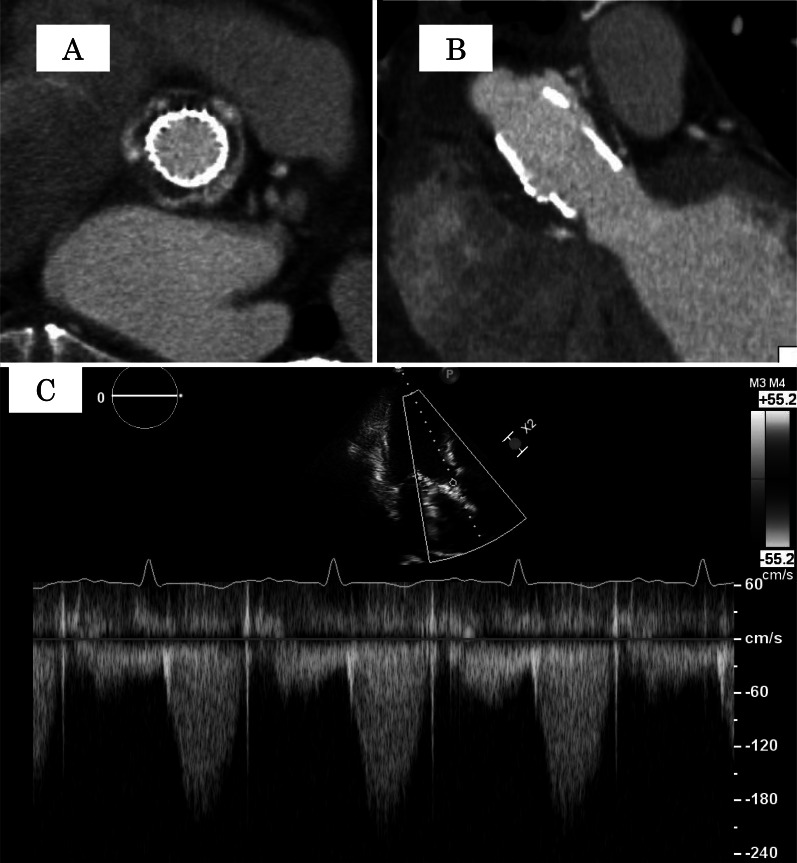
Postoperative CT scan and echocardiography. A postoperative CT scan demonstrates that the sutureless valve adequately expanded at the annulus level (**A** short axis view, **B** long axis view). The pressure gradient through the valve was ten mmHg, and no paravalvular leakage was observed (**C**)

The CPB was weaned off without any concerns. The specimen attached to the bioprosthetic valve was submitted to the microbiology laboratory and was consistent with *Candida parapsilosis*. As a postoperative antifungal regimen, intravenous 125 mg of Amphotericin B and 100 mg of Micafungin sodium hydrate were administered for six weeks after the redo operation. The postoperative course was uneventful, and there was no recurrence of the fungal infection. A postoperative CT scan revealed a sufficiently expanded inflow ring on the annulus. Moreover, transthoracic echocardiography showed no paravalvular leakage and 10 mmHg as the mean pressure gradients through the sutureless valve (Fig. 4A–C). The patient has been followed up in our outpatient clinic with 200 mg of oral itraconazole for life, and the recurrent fungal infection has not been observed for 1 year.

## Discussion

The sutureless valve (Perceval™) has been widely used in various clinical settings, such as isolated, combined, or redo AVR. Concistre et al. reported the outcomes of Perceval™ from a real-world registry [9]. They reported satisfactory data, with an early mortality rate of 0.3%. Regarding midterm outcomes, the 5-year follow-up survival rate was 78.82%, and the 5-year freedom from valve-related major adverse events (death, reintervention, and stroke) was 96.93%. Dhanekula et al. reported a small case series with sutureless valves for redo AVR operations [10]]. This cohort included four cases of prior Bentall procedures. Despite an excellent outcome, with a 30-day mortality rate of 4.5%, the pacemaker implantation rate for the sutureless valve was higher (9.1%) than that of the primary aortic valve operation with Perceval™ valve. In some cases, the implanted Perceval™ valve size was smaller than the explanted valve size in this study. In our case, the inner diameter of the implanted sutureless valve was also smaller than that of the explanted valve by 3 mm; however, the pressure gradient was better in the sutureless valve than in the explanted valve.

Regarding the diagnosis of graft infection, we utilized a PET scan, TEE, and multiple blood cultures to rule out graft infection. However, the PET scan could not completely exclude graft infection due to its low spatial resolution. If vegetation was found attached to the artificial graft or if fluid accumulation around the graft was found, which strongly suggested the graft infection, replacing the artificial graft might be unavoidable.

Technically, several pitfalls are associated with deploying the Perceval™ valve after the modified Bentall procedure. The initial incision on the vascular graft should be made longitudinally from the top of the graft towards the non-coronary sinus to avoid injury to both coronary ostia, terminating 2.5 to 3.0 cm above the sewing ring. Inappropriate sizing and the coronary obstruction due to the wavy stent frame could be common issues in this setting. In terms of sizing, a composite graft anastomosed at the level of the aortic annulus occasionally seemed shrunk owing to the mattress or continuous sutures, and the reimplanted valve resulted in a smaller valve than expected before the operation. Hence, preoperative CT measurement is crucial to ensure accurate valve size. Appreciating the distance between the annulus and coronary ostium is essential to prevent obstruction of the coronary orifice by the wavy stent frames. Structurally, these wavy stents are positioned slightly off-center; therefore, the initial guiding suture should be placed just below the left coronary ostium to ensure clearance. Subsequently, the other two guiding sutures should be placed equidistantly at 120° from the first stitch, referring to the marker of the proper valve sizer. In the Bentall procedure, the right coronary artery is usually anastomosed at a higher location than the normal position to avoid coronary kinking. Therefore, it is unlikely that the wavy stent frames will obstruct the right coronary orifice in this setting. After valve deployment, ballooning at 4 atm for 30 s to appress the inflow ring to the annulus would not suffice because the annulus with the vascular graft would be uneven. In this case, additional ballooning was performed at 5 atm for 15 s to compress the inflow ring to the annulus tightly.

In conclusion, a sutureless valve can facilitate a more straightforward and minimally invasive reoperative procedure. The size and appropriate position of the sutureless valve should be scrutinized using CT when it is applied after the modified Bentall procedure.

## Data Availability

All data generated or analyzed during this study are included in this article.

## Abbreviations

CT: Computed tomography
CABG: Coronary artery bypass grafting
RCA: Right coronary artery
CPB: Cardiopulmonary bypass
VA-ECMO: Venoarterial extracorporeal membrane oxygenation
POD: Postoperative day
PET: Positron emission tomography
TEE: Transesophageal echocardiography
SVG: Saphenous vein graft
AVR: Aortic valve replacement
PVL: Perivalvular leakage

## References

[1] Bentall H, De Bono A. A technique for complete replacement of the ascending aorta. Thorax. 1968;23(4):338–9. 5664694 10.1136/thx.23.4.338 PMC471799

[2] Brown JA, Serna-Gallegos D, Kilic A, Longo S, Chu D, Navid F, et al. Outcomes of reoperative aortic root surgery. J Thorac Cardiovasc Surg. 2023;166(3):716–24. 34776246 10.1016/j.jtcvs.2021.09.060

[3] Leontyev S, Borger MA, Davierwala P, Walther T, Lehmann S, Kempfert J, et al. Redo aortic valve surgery: early and late outcomes. Ann Thorac Surg. 2011;91(4):1120–6. 21276956 10.1016/j.athoracsur.2010.12.053

[4] Jassar AS, Desai ND, Kobrin D, Pochettino A, Vallabhajosyula P, Milewski RK, et al. Outcomes of aortic root replacement after previous aortic root replacement: the “true” redo root. Ann Thorac Surg. 2015;99(5):1601–9. 25754965 10.1016/j.athoracsur.2014.12.038

[5] Michielon G, Salvador L, Da Col U, Valfrè C. Modified button-Bentall operation for aortic root replacement: the miniskirt technique. Ann Thorac Surg. 2001;72(3):S1059–64. 11565727 10.1016/S0003-4975(01)02975-7

[6] Yan TD. Mini-Bentall procedure: the “French cuff” technique. Ann Thorac Surg. 2016;101(2):780–2. 26777944 10.1016/j.athoracsur.2015.06.092

[7] Urbanski PP, Hacker RW. Replacement of the aortic valve and ascending aorta with a valved stentless composite graft: technical considerations and early clinical results. Ann Thorac Surg. 2000;70(1):17–20. 10921675 10.1016/S0003-4975(00)01482-X

[8] Yakut C. A new modified Bentall procedure: the flanged technique. Ann Thorac Surg. 2001;71(6):2050–2. 11426805 10.1016/S0003-4975(01)02439-0

[9] Concistré G, Baghai M, Santarpino G, Royse A, Scherner M, Troise G, et al. Clinical and hemodynamic outcomes of the Perceval sutureless aortic valve from a real-world registry. Interdiscip Cardiovasc Thorac Surg. 2023;36(6):ivad103. 37307090 10.1093/icvts/ivad103 PMC10281856

[10] Dhanekula AS, Nishath T, Aldea GS, Burke CR. Use of a sutureless aortic valve in reoperative aortic valve replacement. JTCVS Tech. 2022;1(13):31–9. 10.1016/j.xjtc.2022.02.025 PMC9196321 35711205

